# Postspinal Surgery Hydrocephalus, Intraventricular Hemorrhage, and Multidrug-Resistant Ventriculitis: A Fatal Cascade

**DOI:** 10.1055/a-2798-9840

**Published:** 2026-02-09

**Authors:** Zeinab El Mawla, Abbas Shoeib, Zahraa Shamas, Ahmad Awde, Ali Raad

**Affiliations:** 1 Department of Pulmonary and Critical Care, Faculty of Medical Sciences, Lebanese University, Hadat, Lebanon; 2 Department of Neurology, Faculty of Medical Sciences, Lebanese University, Hadat, Lebanon; 3 Department of General Medicine, Faculty of Medical Sciences, Lebanese University, Hadat, Lebanon; 4 Department of Neurosurgery, Rassoul Aazam Hospital, Beirut, Lebanon; 5 Pulmonary and Critical Care Division, Rassoul Aazam Hospital, Beirut, Lebanon

**Keywords:** spinal surgery, hydrocephalus, intraventricular hemorrhage, ventriculitis, carbapenem-resistant *Escherichia coli*, intrathecal antibiotics, neurosurgical complications

## Abstract

**Background:**

Hydrocephalus, intraventricular hemorrhage (IVH), and ventriculitis are rare but devastating complications following spinal surgery. Their coexistence significantly worsens prognosis, especially when multidrug-resistant pathogens are involved.

**Case Presentation:**

A 59-year-old man with a C7 fracture and complete paraplegia underwent anterior and posterior cervical spine surgeries complicated by cerebrospinal fluid (CSF) leakage. He later developed hydrocephalus and IVH, requiring external ventricular drainage. Shortly thereafter, fever and altered consciousness occurred, and pus was observed in the drain. CSF analysis confirmed carbapenem-resistant *Enterobacteriaceae* (CRE) ventriculitis, specifically caused by *Escherichia coli* . Treatment with intravenous meropenem, vancomycin, and escalation to intrathecal colistin and tigecycline achieved microbiological clearance. Despite this, neurological recovery was poor, and the patient succumbed 27 days after readmission.

**Conclusion:**

This report emphasizes the interplay of mechanical and infectious complications after spinal surgery, highlighting the need for early recognition, aggressive combined therapy, and preventive strategies to improve outcomes.

## Introduction

Cervical spine trauma and its surgical management carry risks of both mechanical and infectious complications. Hydrocephalus and intraventricular hemorrhage (IVH) following spinal surgery are rare but have been described in association with incidental durotomy, CSF leakage, and alterations in intracranial dynamics. 1 2 3 The underlying mechanisms often involve disruption of CSF absorption, venous hypertension, or secondary hemorrhage, which can compromise neurological outcomes. 4 5

In addition to these mechanical sequelae, neurosurgical patients are at heightened risk for central nervous system infections, particularly in the presence of indwelling devices such as external ventricular drains (EVDs). Postneurosurgical meningitis and ventriculitis represent major sources of morbidity and mortality, with incidence and severity heightened by multidrug-resistant Gram-negative organisms. 6 7 8 Risk factors include the duration of catheter placement, IVH, and impaired host defenses. 9

Carbapenem-resistant *Enterobacteriaceae* (CRE) pose a unique therapeutic challenge, as conventional systemic antibiotics often fail to achieve sufficient CSF concentrations. In this case, the infectious process was driven by a CRE *E. coli* , which likely colonized the patient during his initial prolonged intensive care unit (ICU) stay and subsequently infected the central nervous system via the EVD or through the persistent CSF leak pathway.

For this reason, intraventricular or intrathecal antibiotic administration, particularly colistin, has become a last-line strategy for treatment of multidrug-resistant ventriculitis. 10 11 12 Despite advances in therapy, mortality remains high, particularly in elderly patients and those with severe neurological compromise. 6

The following report describes a patient with cervical spine trauma who developed hydrocephalus, IVH, and ultimately CRE ventriculitis after spinal surgery, illustrating the interaction between mechanical complications and multidrug-resistant infection.

## Case Presentation

**Fig. 1:**
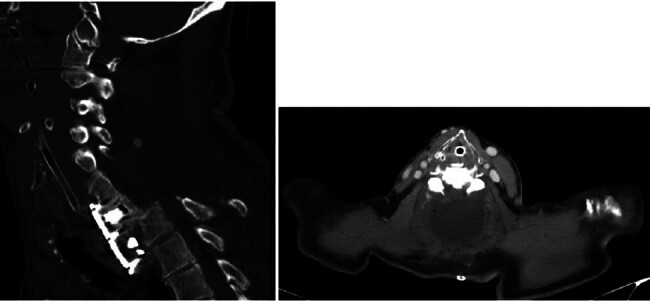
CT scan of the neck demonstrated a displaced C7–T1 segment with cerebrospinal fluid (CSF) collection around the site.

A 59-year-old man, a heavy smoker with a history of hypertension, sustained a traumatic cervical spine injury following a motor vehicle accident. Imaging revealed a C7 vertebral fracture associated with complete paraplegia. The patient initially underwent posterior cervical spine surgery at C6 to C7 and was subsequently transferred to the ICU postoperatively at another hospital. He was later transferred to our hospital intubated for continuation of care. CT imaging demonstrated a displaced C7 to T1 segment with cerebrospinal fluid (CSF) collection around the surgical site ( Fig. 1 ), indicating a significant incidental durotomy and CSF leak that occurred during the index operation at the outside facility. The operative report from the referring institution confirmed a difficult posterior decompression with a noted dural tear that was primarily repaired but remained a source of persistent leakage. CT imaging also showed displaced screws from the initial C6 to C7 fixation, which were encroaching upon the spinal canal and contributing to the persistent CSF collection, prompting consideration for surgical intervention. However, the patient exhibited autonomic dysfunction, including bradycardia and hypotension upon repositioning or sedation, which contraindicated prone positioning. Consequently, an anterior surgical approach was selected to remove displaced screws, decompress the spinal cord, and perform vertebral body removal, during which the CSF collection was evacuated. One week later, the patient underwent posterior cervical surgery in the prone position.

Postoperatively, the patient showed neurological improvement in the ICU, regaining some sensation and movement in the upper limbs and partial sensory recovery in the lower limbs.

**Fig. 2:**
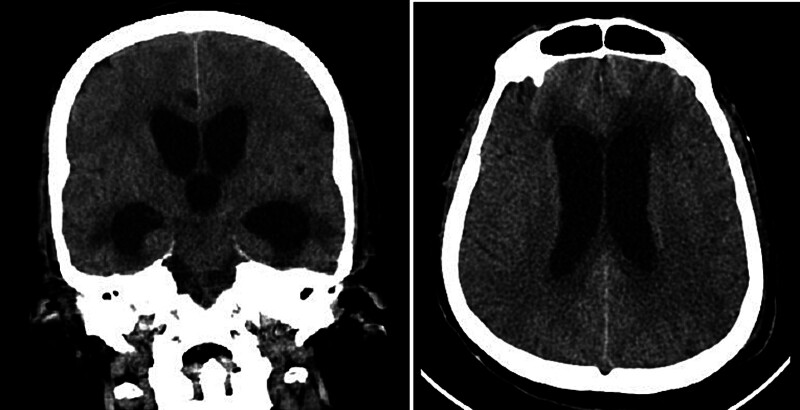
CT imaging of the brain showed hydrocephalus.

**Fig. 3:**
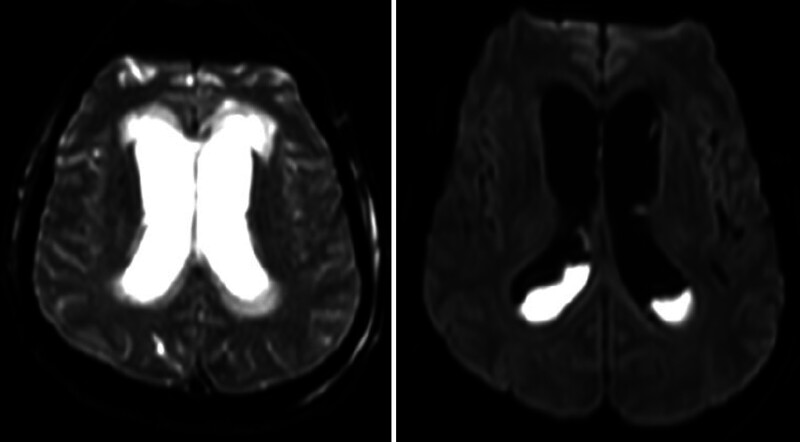
MRI brain confirmed progressive hydrocephalus with intraventricular hemorrhage.

During his ICU stay, he developed ventilator-associated pneumonia, which was successfully treated, allowing for extubation and discharge home after 1 month. Two days later, he was readmitted with decreased level of consciousness and desaturation, necessitating reintubation. Neurological assessment revealed myoclonic movements, and urgent CT imaging of the brain demonstrated hydrocephalus ( Fig. 2 ). Magnetic resonance imaging confirmed progressive hydrocephalus with IVH, likely resulting from the chronic CSF hypotension and subsequent intracranial venous congestion caused by the persistent spinal CSF leak ( Fig. 3 ), and an urgent EVD was placed. The patient showed slight improvement but developed fever and altered consciousness within 24 hours of EVD placement, with frank pus observed in the EVD almost immediately, suggesting that the patient may have already had a subclinical or developing ventriculitis seeded from the chronic spinal infection site, which became florid upon the introduction of the EVD and the shift in CSF dynamics. CSF analysis revealed bacterial meningitis Table 1 .

**Table 1:** CSF analysis and cultures

Date	CSF WBC (cells/µL)	RBC	Neutrophils (%)	Glucose (mg/dL)	Protein (mg/dL)	LDH	CSF Culture
June 30, 2025	177	0	84.1	29	65.2	1211	Few *E. coli* CRE
July 4, 2025	33500	49,000	98	15	220.1	4250	Moderate *E. coli* CRE
July 7, 2025	6481	6,000	98	16	269.4	945	No growth
July 10, 2025	1400	3,000	94.6	7	1999.9	945	No growth
July 15, 2025	215	6,000	90.7	26	107	—	No growth

On June 30, 2025, CSF analysis showed 177 white blood cell count (WBC)/µL with 84.1% neutrophils, glucose 29 mg/dL, protein 65.2 mg/dL, and lactate dehydrogenase (LDH) 1211 U/L; culture grew few colonies of CRE *E. coli* . Over the following week, serial CSF studies demonstrated worsening inflammation. On July 4, 2025, WBC increased to 33,500/µL with 98% neutrophils, glucose decreased to 15 mg/dL, protein rose to 220.1 mg/dL, and LDH reached 4250 U/L, with culture showing moderate CRE growth. Despite treatment with intravenous meropenem and vancomycin, CSF inflammation persisted, prompting escalation to intravenous and intrathecal colistin and tigecycline, in addition to continued meropenem. On July 7, 2025, WBC decreased to 6,481/µL with 98% neutrophils, glucose 16 mg/dL, protein 269.4 mg/dL, and no bacterial growth. On July 10, 2025, WBC was 1,400/µL, glucose 7 mg/dL, and protein 1,999.9 mg/dL, with cultures remaining negative. By July 15, 2025, after intravenous and intrathecal colistin, meropenem, and tigecycline therapy, CSF analysis revealed 215 WBC/µL, glucose 26 mg/dL, protein 107 mg/dL, and no bacterial growth.

Although cultures eventually cleared, the patient's neurological prognosis remained poor due to complications from hydrocephalus, IVH, and the prolonged infectious process. The patient ultimately passed away 27 days after readmission.

## Discussion

The clinical course illustrates the convergence of rare but devastating postoperative complications following cervical spine surgery. Hydrocephalus, though unusual in this context, has been previously reported as a consequence of IVH or CSF leakage.

Pathophysiologically, intraventricular blood interferes with normal CSF reabsorption through obstruction of arachnoid granulations, as well as through inflammatory processes that exacerbate ventricular dilation. 2 4 In the presented patient, hydrocephalus manifested with progressive neurological decline, and neuroimaging confirmed ventricular enlargement with IVH. Prompt placement of an EVD was lifesaving, aligning with current recommendations for management of acute hydrocephalus. 8

However, the presence of an EVD carries substantial infection risk. A systematic review demonstrated that duration of catheter placement and IVH are independent predictors of ventriculitis. 9 In this setting, bacterial meningitis and ventriculitis manifest with purulent drainage, profound CSF pleocytosis, hypoglycorrhachia, and elevated protein levels. The progression from initial modest inflammation to fulminant ventriculitis in this patient underscores the rapid evolution of catheter-associated infections.

The rapid development of florid ventriculitis suggests a mechanistic link between the chronic spinal CSF leak and the subsequent intracranial infection. It is hypothesized that the persistent leak created a low-pressure system that facilitated the retrograde migration of pathogens from the surgical site or the environment, which then proliferated rapidly once the EVD was placed and intracranial dynamics were further altered.

Microbiological findings revealed CRE, an increasingly encountered pathogen in neurosurgical units. CRE infections pose significant therapeutic challenges due to resistance to most available β-lactams and poor penetration of systemic antibiotics into CSF. Standard intravenous therapy is often inadequate, necessitating alternative strategies. Intraventricular and intrathecal delivery of colistin has been supported by several reviews as a salvage therapy for multidrug-resistant Gram-negative meningitis, often achieving microbiological clearance when systemic therapy fails. 10 11 Tigecycline, despite limited CSF penetration, has been used as adjunctive therapy in refractory infections. The combination of intravenous meropenem with intrathecal colistin and tigecycline in this case led to eventual culture clearance, consistent with published outcomes in resistant infections.

Nevertheless, clearance of infection did not translate into neurological recovery. Persistent hydrocephalus, the inflammatory burden of ventriculitis, and the sequelae of IVH together contributed to irreversible damage. IVH itself is associated with poor prognosis due to secondary injury cascades and mechanical obstruction of CSF pathways. 13 14 Mortality in patients with postneurosurgical meningitis remains high, particularly when multidrug-resistant organisms are involved, as highlighted in recent large cohort studies. 6

The clinical trajectory emphasizes the interdependence of surgical, mechanical, and infectious factors. While aggressive antimicrobial strategies can achieve microbiological control, prevention remains the most effective strategy. Minimizing intraoperative CSF leakage, limiting duration of EVD use, and adhering to strict infection control protocols are crucial measures to reduce the risk of these devastating complications. 7 9

## Conclusion

Hydrocephalus and IVH represent rare but serious complications after spinal surgery, often requiring urgent CSF diversion. The use of ventricular drains, although essential, predisposes patients to infection, particularly with multidrug-resistant organisms such as CRE. Even with aggressive systemic and intrathecal therapy, outcomes remain poor when infection is superimposed on severe neurological injury. This report underscores the need for early recognition, rapid intervention, and multidisciplinary management, while highlighting prevention strategies as the cornerstone of reducing morbidity and mortality in neurosurgical patients. The authors testify that this manuscript is the result of original clinical observation and human-authored analysis, complying with all ethical and journal standards regarding the use of assistive technologies.
